# Malaria Trends, Burden, Seasonal Variation, and Interventions in Western Tigray, Ethiopia

**DOI:** 10.1155/jotm/3197517

**Published:** 2025-12-12

**Authors:** Getachew Belay Kassahun, Amanuel Mesele Berhe, Merhawi Alemu Brhanu, Brhane Berhe Aregawi

**Affiliations:** ^1^ Department of Medical Laboratory Sciences, College of Medicine and Health Sciences, Adigrat University, Adigrat, Ethiopia, adu.edu.et; ^2^ Department of Public Health, College of Medicine and Health Sciences, Adigrat University, Adigrat, Ethiopia, adu.edu.et

**Keywords:** burden, intervention, malaria, seasonal variation, Tigray, trend

## Abstract

**Background:**

Despite being preventable and treatable, malaria continues to have a devastating impact on people’s health and livelihoods around the world. In Ethiopia, it is one of the three leading causes of hospital admission and mortality. Therefore, the aim of this study was to assess the trend, burden, seasonal variations, and interventional assessment of malaria in Western Tigray, Ethiopia, from 2011 to 2019.

**Methods:**

A hospital‐based retrospective cross‐sectional study was carried out to determine trend, burden, seasonal variations, and interventional assessment of malaria in Kahsay Abera General Hospital, Western Tigray, North Ethiopia, from 2011 to 2019. All recorded microscopically confirmed malaria cases in the Health Management Information System of the hospital were carefully taken and analyzed. Also, malaria intervention activities applied in the area were assessed using a checklist, personal communication with hospital administrators, and observations. All data of malaria cases were entered and analyzed using Microsoft Office Excel and presented in tables and figures.

**Results:**

A total of 36,438 malaria cases with 50 (0.14%) hospital mortality and 2016 (5.5%) hospital admissions were recorded from 2011 to 2019. *Plasmodium falciparum,* with 22,621 cases (62.1%), was the predominant malaria species identified. The highest hospital malaria death was observed in the age group ≥ 15 years, with 38 cases (0.10%), and the highest hospital deaths occurred during October–December, with 21 cases (0.06%) of the total confirmed malaria cases. Although the fluctuating trend of malaria cases, with no shift in species, was statistically significant (*p* = 0.001) over the study period, the trend in hospital mortality due to malaria was not statistically significant (*p* = 0.62).

**Conclusions:**

Despite Ethiopia’s notable progress in malaria control, the disease remains a major health problem with fluctuating annual trends.

## 1. Introduction

Despite various control strategies, malaria continues to have a devastating global impact on health and livelihoods [1, 2]. Globally, in 2023, there were nearly 263 million estimated malaria cases across 83 malaria‐endemic countries. Since 2020, the number of estimated malaria cases has steadily increased, with most of this rise occurring in the African region (89.7%). Ethiopia was the country primarily contributing to the increase in malaria cases between 2022 and 2023, with an additional 4.5 million cases [3]. In most countries where malaria is endemic, the disease disproportionately affects poor and disadvantaged people, who have limited access to health care and cannot afford appropriate treatment [4].

The burden of malaria is very severe in Ethiopia where it had been the major cause of morbidity and mortality for years [4]. It leads more than 50 million people at risk [5], and 4–5 million people are affected annually in the country [5]. It has consistently been one of the three principal causes of morbidity and mortality. It was also a leading cause of outpatient visits, admissions, and deaths in the country, accounting for over 20% of deaths at all ages [6]. Although Ethiopia has achieved remarkable progress in fighting the disease following the national guideline, about 5–6 million annual confirmed malaria cases were observed [5]. Cessation of malaria control measures due to war and pandemic disease outbreaks, along with other challenges, were reviewed as possible contributing factors to ongoing malaria burden. These include climate change, suboptimal uptake of antimalarial intervention, poor data quality and utilization, vector insecticide resistance, low coverage of malaria impeding service, poor access to health care, poor follow‐up on malaria control measures, increasing trend in internal displacement of people, and limited funding (financial and human resources) [5–8].

In Tigray, although the proportion of total OPD visits, admissions, and deaths decreased, malaria remains the second leading cause of both outpatient and inpatient morbidity [9]. Therefore, this study aimed to assess the trend, burden, seasonal variations, and interventional assessment of malaria in Western Tigray, Ethiopia, from 2011 to 2019. The study also highlights regional and zonal efforts to control and prevent malaria.

### 1.1. Study Area, Period, and Design

A hospital‐based retrospective cross‐sectional study was conducted at Kahsay Abera General Hospital, Humera, Tigray, Ethiopia, covering the period from 2011 to 2019. Humera town lies about 984 km (611 miles) northwest of Addis Ababa, the capital, and 515 km (320 miles) west of Mekelle in the Tigray Region. The town sits at an elevation ranging from 585 m above sea level and experiences a hot semiarid climate. Year round climate is generally mild to dry, with average annual rainfall between 400 and 600 mm, predominantly occurring from June to September [10]. According to the 2007 Census by the Central Statistical Agency of Ethiopia (CSA), the town’s total population was 21,653, comprising 11,395 men and 10,258 women [11]. Additionally, the region’s agricultural expansion, which includes mechanized farming and irrigation, has increased the presence of breeding sites, making both permanent and migrant agricultural workers vulnerable to malaria disease during peak farming seasons. Kahsay Abera General Hospital is the second hospital in Western Tigray and serves as large catchment area that includes people from various regions of Ethiopia, Eritrea, and Sudan, in addition to the local zonal population. Currently, the hospital provides emergency, inpatient, outpatients, and referral services.

### 1.2. Source of Data

In Kahsay Abera General Hospital, malaria cases were confirmed, with Giemsa stain microscopically as diagnostic tool, in the laboratory department. Monthly confirmations were then reported from laboratory to the hospital’s Health Management Information System (HMIS) department. Data were stored in the HMIS database by year, month, sex, and age category. The present study included microscopically confirmed malaria species data recorded in HMIS office from 2011 to 2019 at Kahsay Abera General Hospital. In addition, Interventions applied in the study area were assessed by checklists, communication with hospital administrators, and observations.

### 1.3. Eligibility Criteria

Data in the HMIS contained full information such as year, month, sex, age category, and confirmed malaria cases which were included. However, any data not properly recorded or missing were excluded from the analysis.

### 1.4. Sample Size Determination

All 36,604 microscopically confirmed malaria reports registered in HMIS from 2011 to 2019 were included in the final analysis, as there were no incomplete or missed data.

• **Dependent variable:** Trend, hospital admissions, and number of hospital mortality rate.
• **Independent variables:** Demographic data (age and sex), year, interventions of malaria, and seasonal variability.

### 1.5. Data Collection Procedure

Nine‐year malaria data (2011–2019) were collected from Kahsay Abera General Hospital HMIS department. Secondary data of malaria confirmed cases were collected in Microsoft Office Excel 2010 spreadsheet. Demographic data and the species identified by year and month were collected by five trained Medical Laboratory Technologists, under the supervision of the principal investigator and HMIS expert. A 2‐day training was provided to the technologists to understand the study objective and data requirements. Moreover, seasonal confirmed cases, hospital admission, and mortality of malaria were collected. Interventions were assessed using a checklist, communication with hospital administrators, and observations. The interventions included community awareness, provision of insecticide‐treated bed nets, budget allocation, and trainings for health professionals by government and nongovernmental organizations.

### 1.6. Data Quality Control

To assure the quality of data, we first assessed the completeness of HMIS malaria data in Kahsay Abera General Hospital. The data collectors were adequately informed about the data collection process. Moreover, the completeness and consistency of the data were checked by double entry and filter in Microsoft Office Excel 2010.

### 1.7. Data Analysis

All data taken from HMIS were checked for completeness and analyzed using Microsoft Office Excel 2010. Descriptive analysis, charts, and linear regression were statistical packages used to analyze the findings. The data were summarized and presented in the form of tables and figures. Monthly, yearly, and quarterly data of malaria cases and mortality were described in the table form. The changes in malaria cases and mortality over time were calculated using linear regression, with *p* values < 0.05 considered statistically significant. In addition, linear regression assumption and model fit were checked, with *R* ^2^ approaching 1 as an indicator of good fit.

### 1.8. Operational Definitions

• **Quarter 1:** Seasonal period from July to September (according to HMIS report of the study area).
• **Quarter 2:** Seasonal period from October to December (according to HMIS report of the study area).
• **Quarter 3:** Seasonal period from January to March (according to HMIS report of the study area).
• **Quarter 4:** Seasonal period from April to June (according to HMIS report of the study area).
• **Hospital mortality rate:**​ The proportion of malaria mortality from the total malaria confirmed cases in the hospital.
• **Trend:** Malaria confirmed cases in years and seasonal period.
• **Burden:** The overall sum of outpatients, inpatients, and death due to malaria case.

### 1.9. Ethical Consideration

Permission letter and ethical clearance were obtained from Research and Ethics Institutional Review Board of Adigrat University (REIB). After discussing the purpose and relevance of the study, we have got verbal and written permission letter from the administration of Kahsay Abera General Hospital before staring data collection.

## 2. Results

### 2.1. Overall Trend and Burden of Malaria Cases in Kahsay Abera General Hospital From 2011 to 2019

**Figure 1:**
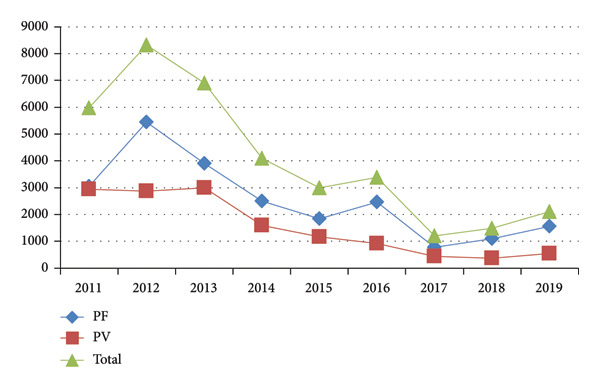
Trend of malaria cases confirmed microscopically by year in Kahsay Abera Hospital, Ethiopia, from 2011 to 2019.

**Figure 2:**
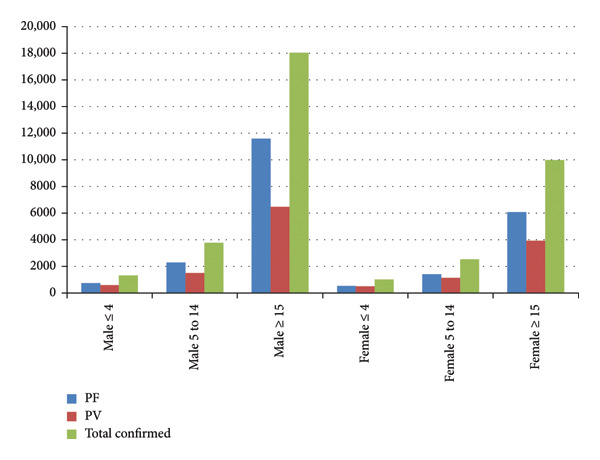
Number of malaria cases confirmed microscopically distributed by age and sex in Kahsay Abera Hospital, Ethiopia, from 2011 to 2019.

A total of 36,438 microscopically confirmed malaria cases were recorded between 2011 and 2019. The annual mean + standard deviation of microscopically confirmed malaria cases was 4048.6 + 2501.9, respectively. The minimum and maximum number of microscopically confirmed malaria cases was reported in 2017, 1206 (3.3%), and in 2012, 8313 (22.8%), respectively (Table 1). Malaria cases varied considerably from year to year, with peaks observed in 2012 and declines in subsequent years (Table 1 and Figure 1). Both *Plasmodium falciparum* and *Plasmodium vivax* were reported each year, with no shift in species, accounting for 22,621 (62%) and 13,817 (38%), respectively. The fluctuating trend of malaria cases over time was statistical significant with *p* value of 0.001 (Table 1), *R* ^2^ = 0.78, and slope of −801.47. The highest prevalence of microscopically confirmed malaria was observed during the seasonal period of Quarter 2 (October–December) which accounted for 48.8% (*N* = 17,780) of the total malaria cases (Table 1). The most affected groups were males (63.2%) (*N* = 23,046) and the age group of ≥ 15 years (77%) (*N* = 28,052) (Table 1 and Figure 2). The overall hospital admitted patients due to malaria case were 2016 (5.5%) of the total confirmed malaria cases. The highest hospital admission per year was recorded in 2017 (*N* = 221, 18.3%), and the lowest admission was recorded in 2011 (*N* = 137, 2.3%). The overall hospital mortality rate of malaria was 0.14% (*N* = 50) of the total confirmed malaria cases over the study period. The highest hospital mortality rate per year was recorded in 2017 at 0.41% (*N* = 5), while the lowest hospital mortality rate was recorded in 2012 at 0.05% (*N* = 4) (Table 1).

**Table 1:** Trend of microscopically confirmed malaria cases and burden of malaria cases from 2011 to 2019 in Kahsay Abera Hospital, Ethiopia.

Year	Malaria cases by season				Sex		Age in years						PS		TCC	H.A.P, *N*, %	Mortality, *N*, %	*p* value of TCC
	Q1, *N*, %	Q2, *N*, %	Q3, N, %	Q4, *N*, %	Male, *N*, %	Female, *N*, %	< 5		5–14		≥ 15		PF, *N*, %	PV, *N*, %	Total, *N*, %			
							M, *N*, %	F, *N*, %	M, *N*, %	F, *N*, %	M, *N*, %	F, *N*, %						
2011	1934, 32.4	2868, 48	932, 15.6	231, 3.8	3927, 65.8	2047, 34.3	229, 3.8	130, 2.1	998, 16.7	509, 8.5	2700, 45.2	1408, 23.6	3030, 50.7	2935, 49.2	5965, 16.4	137, 2.3	5, 0.08	0.001^∗^
2012	2531, 30.5	3441, 41.4	1248, 15	1093, 13.1	5190, 62.4	3123, 37.6	266, 3.2	244, 2.9	1128, 13.6	733, 8.8	3796, 45.7	2146, 25.8	5451, 65.6	2862, 34.4	8313, 22.8	293, 3.5	4, 0.05	
2013	2133, 30.9	3316, 48.1	979, 14.2	475, 6.8	4376, 63.4	2527, 36.6	334, 4.8	220, 3.2	429, 6.2	344, 4.9	3613, 52.3	1963, 28.4	3905, 56.5	2998, 43.4	6903, 18.9	183, 2.6	7, 0.11	
2014	814, 19.9	2308, 56.5	657, 16.1	308, 7.5	2553, 62.5	1534, 37.5	73, 1.8	98, 2.4	214, 5.2	172, 4.2	2266, 55.4	1264, 30.9	2489, 60.9	1598, 39.1	4087, 11.2	231, 5.6	4, 0.1	
2015	1238, 41.4	1296, 43.3	270, 9.1	189, 6.3	1799, 60.1	1194, 39.8	146, 4.9	111, 3.7	255, 8.5	239, 7.9	1398, 46.7	844, 28.2	1836, 61.3	1157, 38.6	2993, 8.2	200, 6.7	4, 0.13	
2016	858, 25.4	1826, 54.0	467, 13.8	230, 6.8	1984, 58.7	1397, 41.3	63, 1.8	48, 1.4	385, 11.4	241, 7.1	1536, 45.4	1108, 32.8	2463, 72.8	918, 27.2	3381, 9.3	459, 13.5	10, 0.29	
2017	558, 46.3	396, 32.8	168, 13.9	84, 6.9	715, 59.3	491, 40.7	32, 2.6	49, 4.1	123, 10.2	80, 6.6	560, 46.4	362, 30.0	773, 64.1	433, 35.9	1206, 3.3	221, 18.3	5, 0.41	
2018	392, 26.4	924, 62.2	157, 10.6	13, 0.9	1010, 67.9	467, 31.4	43, 2.9	25, 1.7	89, 5.9	50, 3.4	878, 59.1	392, 26.4	1112, 74.8	374, 25.2	1486, 4.1	137, 8.7	6, 0.40	
2019	214, 10.2	1405, 66.8	395, 18.8	90, 4.3	1492, 70.9	612, 29.1	54, 2.6	41, 1.9	116, 5.5	75, 3.6	1322, 62.8	496, 23.6	1562, 74.2	542, 25.8	2104, 5.8	155, 9.2	5, 0.24	

Total	10672, 29.3	17780, 48.8	5273, 14.5	2713, 7.4	23046, 63.2	13392, 36.7	1240, 3.4	966, 2.6	3737, 10.2	2443, 6.7	18069, 49.6	9983, 27.4	22621, 62.1	13817, 37.9	36438	2016, 5.5	50, 0.14	

*Note: N*, total, %, percentage, M, male, F, female, Q1, Quarter 1, Q2, Quarter 2, Q3, Quarter 3, and Q4, Quarter 4.

Abbreviations: H.A.P, hospital admitted patients; PF, *Plasmodium falciparum*; PS, *Plasmodium* species; PV, *Plasmodium vivax*; TCC, total confirmed cases.

^∗^Statistical significance.

### 2.2. Malaria Intervention Measures and Gaps Identified in the Study Area

Malaria control and preventive measures taken each year from 2011 to 2019 were assessed using a checklist, communication with hospital administrators, and observations. The interventions included community awareness through mass education about malaria control measures in health facilities (2011–2019); provision of insecticide‐treated bed nets to the community (2013–2019); increases in the budget for malaria prevention and control activities by the FMOH, Regional Health Bureau, and various stakeholders (obtained through personal communication) (2005–2013); and different training sessions arranged by nongovernmental organizations, the FMOH, and the Regional Health Bureau on malaria diagnosis (2016–2019). Additionally, case management of *P. falciparum* and *P. vivax* was conducted based on national guidelines (obtained through personal communication and observations) (2004–2019). Furthermore, active malaria surveillance was conducted and reported to the HMIS department of the hospital (2010–2019). Therefore, hospital‐based awareness creation for the community and loss of follow‐up on the application of insecticide‐treated bed nets, despite distributing the available treated bed nets, were identified as gaps.

### 2.3. Hospital Mortality by Demography (Age and Sex) and Seasons

The peak number of hospital deaths and admitted patients was recorded in 2016, with 10 deaths and 459 admitted patients, respectively (Tables 1 and 2). The highest number of hospital malaria deaths was observed among the age group of ≥ 15 years, with 38 deaths (Table 2). Furthermore, the maximum number of hospital deaths occurred in seasonal period Quarter 2, with 21 deaths, while 45 deaths occurred among male patients (Table 2). Despite the fluctuating trend of malaria cases, there were no statistically significant difference of hospital mortality across the study years​ (*p*​ value of 0.62) (Table 2).

**Table 2:** Hospital death of malaria by demography (age and sex), *Plasmodium* species, and seasons from 2011 to 2019.

Years	Mortality by season				Mortality by age			Mortality by sex		Mortality by PS	*p* value
	Q1, *N*%	Q2, *N*%	Q3, *N*%	Q4, *N*%	≤ 4, *N*%	5–14 *N*%	≥ 15 *N*%	M, *N*%	F, *N*%	Total	
2011	2 (0.03)	2 (0.03)	1 (0.02)	—	—	—	5 (0.08)	5 (0.08)	—	5 (0.08)	0.62
2012	2 (0.02)	2 (0.02)	—		—	1 (0.01)	3 (0.04)	4 (0.05)	—	4 (0.05)	
2013	2 (0.03)	2 (0.03)	2 (0.03)	1 (0.01)	4 (0.06)	—	3 (0.04)	6 (0.09)	1 (0.01)	7 (0.11)	
2014	—	3 (0.07)	1 (0.02)	—	1 (0.02)	1 (0.02)	2 (0.05)	4 (0.10)	—	4 (0.10)	
2015	2 (0.07)	2 (0.07)	—	—	1 (0.03)	—	3 (0.10)	3 (0.10)	1 (0.03)	4 (0.13)	
2016	5 (0.15)	3 (0.09)	—	2 (0.06)	1 (0.03)	—	9 (0.27)	10 (0.29)	—	10 (0.29)	
2017	2 (0.17)	1 (0.08)	2 (0.17)	—	2 (0.17)	—	3 (0.25)	4 (0.33)	1 (0.08)	5 (0.41)	
2018	2 (0.13)	4 (0.27)	—	—	—	—	6 (0.40)	5 (0.34)	1 (0.07)	6 (0.40)	
2019	1 (0.05)	2 (0.09)	2 (0.09)	—	—	1 (0.05)	4 (0.19)	4 (0.19)	1 (0.05)	5 (0.24)	
Total	18 (0.05)	21 (0.06)	8 (0.02)	3 (0.01)	9 (0.03)	3 (0.01)	38 (0.10)	45 (0.12)	5 (0.02)	50 (0.14)	

*Note:* “—” = no data, Q1, Quarter 1, Q2, Quarter 2, Q3, Quarter 3, Q4, Quarter 4, *N*, total malaria mortality, %, percentage, M, male, and F, female.

Abbreviation: PS, *Plasmodium* species.

## 3. Discussion

Examining and assessing the current malaria status is an important component of malaria control, as it measures the achievement of ongoing interventions and guides the planning of prospective control and elimination efforts. Hence, the aim of the current study was to assess the trend, burden, seasonal variations, and interventions of malaria in Western Tigray, North Ethiopia, from 2011 to 2019. The study recorded a fluctuating trend of malaria cases (Figure 1 and Table 1), which is consistent with studies conducted in different parts of Ethiopia [7, 9, 12–14]. The year‐to‐year variation recorded in different countries might be due to the interplay of various factors, including the intervention measures undertaken, climate conditions, policy variations from region to region within the same country, diagnostic techniques used, the skills of laboratory personnel, endemicity of the study areas, and types of malaria control measures applied.

According to the current study, *Plasmodium falciparum* was the predominant malaria species identified. This is similar to the studies conducted in different parts of Ethiopia [10, 13, 15–18]. This might be due to the fact that *Plasmodium falciparum* undergoes five stages of development in 9–12 days [19]. Thus, the longest maturation time and remaining infectious for several days compared with other malaria parasites could be the possible reason for predominancy. Besides, the predominancy might be due to vector preference, geographic distribution, drug resistance, biological difference, altitude, and environmental suitability. Likewise, the current study showed that the highest number of confirmed malaria species was present in Quarter 2 seasonal period. This agrees with the studies done in different parts of Ethiopia [4, 8, 11, 12, 15, 19, 20]. However, the findings differ from the results of the study conducted at South Omo Zone, Ethiopia, which was conducted from April to June seasonal period [18]. Climate change has been noticed worldwide with impact on rainfall, temperature, and humidity. Several studies have demonstrated the influence of climate change in the prevalence of malaria cases [21]. Therefore, the difference might be due to the climate change and high population movement to Humera during October–December for agricultural activities that could influence the number of cases.

The current study also revealed that males aged ≥ 15 years old were more affected by malaria cases. This is parallel to the study reported in different parts of Ethiopia [5, 9, 13–16, 18, 20, 22, 23]. A study in sub‐Saharan Africa predicted that in the near future, malaria may become an adult disease, shifting from children to older age due to successfully targeted intervention programs at the very young age along with various social, cultural, economical, and behavioral factors, which increase exposure and reduce the uptake and effectiveness of interventions in the older age groups [24]. High number of adult male population movement to the study area for agricultural work might be a cause for increased confirmed malaria species among age group of 15 years old and above.

The overall hospital admitted patients and hospital mortality rate during the study period were 2016 (5.5%) and 50 (0.14%), respectively, of the total confirmed malaria cases. The hospital mortality rate increased from 2014 to 2017 and then declined through 2019. The decrease in hospital mortality rate in 2018 and 2019 compared with previous years may be due to the impact of interventions implemented in the study area. These included an increase in the budget by the FMOH, the provision of insecticide‐treated bed nets to the community, facility‐based health education, and changing treatment guidelines.

Furthermore, the peak hospital mortality rate and admission due to malaria case were recorded in 2016. The highest hospital malaria death was observed in males with age group of 15 years old and above. This is comparable with a study done in Kenya and India [25, 26]. This could be due to behavioral factors, occupational risks, migration and mobility, and policy and intervention implications. On the other hand, the highest malaria death occurred in Quarter 2 seasonal period. This might be due to high number of confirmed malaria cases because of population movement for agricultural cultivation in the study area.

## 4. Conclusions and Recommendations

The current study demonstrated that malaria remains a public health burden in the area. The deadly *P. falciparum* appears to be the principal *Plasmodium* species. High hospital admission and mortality occurred among males aged 15 years and older. Additionally, Quarter 2 recorded the highest hospital admission and mortality rates during the seasonal period. Therefore, health decision makers at the national, regional, and zonal levels need to strengthen evidence‐based malaria prevention and control measures to interrupt disease transmission and ultimately reduce malaria cases in the study area.

## 5. Limitation of the Study

This study was hospital‐based and dependent on HMIS data. The study was unable to include prevalence of malaria due to incompleteness of data in the HMIS report. The HMIS data appeared to be incomplete.

**Nomenclature**
HMIS: Health Management Information System
CSA: Central Statistical Agency
SOPs: Standard operating procedures
OPD: Outpatient Department
FMOH: Federal Ministry of Health

## Conflicts of Interest

The authors declare no conflicts of interest.

## Author Contributions

G.B.K.: conceptualization of the study, drafting of the manuscript, data curation, and revision of the manuscript. A.M.B.: data collection. M.A.B.: data collection. B.B.A.: conceptualization of the study, data curation, and revision of the manuscript.

## Funding

No funding was available to conduct the study.

## Data Availability

All the data generated and analyzed are included in the article and available upon request.
